# Social Transfers for Exclusive Breastfeeding (STEB) Intervention in Lao People’s Democratic Republic: Protocol for a Randomized Controlled Trial

**DOI:** 10.2196/54768

**Published:** 2024-05-03

**Authors:** Souliviengkham Sonephet, Sengchanh Kounnavong, Lucienne Zinsstag, Pascale Vonaesch, Somphou Sayasone, Latsamy Siengsounthone, Peter Odermatt, Günther Fink, Jordyn Tinka Wallenborn

**Affiliations:** 1 Lao Tropical and Public Health Institute Vientiane Lao People's Democratic Republic; 2 Department of Epidemiology and Public Health Swiss Tropical and Public Health Institute Allschwil Switzerland; 3 Department of Fundamental Microbiology University of Lausanne Lausanne Switzerland; 4 University of Basel Basel Switzerland

**Keywords:** breastfeeding, lactation, human milk, breastmilk, child, infant, health, growth and development, cash transfer, incentive, intervention

## Abstract

**Background:**

Children in Lao People’s Democratic Republic (Lao PDR) receive suboptimal nutrition because of low breastfeeding rates, undermining their developmental potential. While major public health campaigns have attempted to increase breastfeeding rates, they have been largely unsuccessful. One explanation for these unsuccessful interventions is the economic and financial constraints faced by mothers. A potential solution for alleviating these pressures is providing social transfers to support breastfeeding; defined as a cash or in-kind transfer. Capitalizing on key strategies used in previous social transfer programs, we will assess the effectiveness of social transfer intervention for increasing exclusive breastfeeding rates in Vientiane, Lao PDR.

**Objective:**

This study aims to conduct a randomized controlled trial (RCT) designed to assess whether social transfers can increase exclusive breastfeeding rates in Vientiane Capital, Lao PDR.

**Methods:**

A prospective, parallel cluster-RCT was conducted among 300 mothers who recently gave birth and initiated breastfeeding. Enrolling 100 participants for each intervention arm provided us with 80% power to detect an increase in exclusive breastfeeding from the anticipated 21% in the control arm to 40% in either of the 2 intervention arms. Mother-infant dyads were enrolled at approximately 1 month post partum. Follow-up visits will occur at 6 months, 1 year, 2 years, and 3 years post partum; with the ambition to extend the follow-up period. Mother-infant dyads were enrolled between August 2022 and April 2023 with follow-up until 3 years post partum (2026). A local study team comprised of 2 nurses and 2 laboratory technicians is responsible for enrollment and follow-up of participants. Participants were randomly assigned to one of three groups during the baseline, 1-month visit: (1) control group, no social transfer; (2) intervention group 1, an unconditional social transfer at 6 months post partum; and (3) intervention group 2, a social transfer at 6 months post partum conditional upon mothers exclusively breastfeeding. All groups received educational materials supporting mothers to exclusively breastfeed. The primary end point will be exclusive breastfeeding at 6 months post partum. Secondary end points will include exclusive and complementary breastfeeding duration, childhood wasting and stunting, child growth, maternal and infant stress, predictors of early breastfeeding cessation, intestinal inflammation, anemia, maternal weight loss, maternal blood pressure, maternal anxiety, and GRIT personality score. Questionnaires and physical examinations were used to collect information.

**Results:**

As of November 2023, the study has enrolled 300 participants. Study participation is ongoing until December 2026 at minimum. Over the study lifetime, 93% have completed all visits.

**Conclusions:**

We see potential for a long-term program that may be implemented in other low- or lower-middle-income countries with only minor modifications. The RCT will be used as a basis for observational studies and to investigate the impact of human milk on child fecal microbiota and growth.

**Trial Registration:**

ClinicalTrials.gov NCT05665049; https://clinicaltrials.gov/study/NCT05665049

**International Registered Report Identifier (IRRID):**

DERR1-10.2196/54768

## Introduction

Breastfeeding provides important benefits for infants and societies [1,2]. Despite its well-known and widespread benefits, breastfeeding rates remain low in many parts of the world. In Vientiane, the capital of Lao People’s Democratic Republic (Lao PDR), only 21% of children aged 0-5 months were exclusively breastfed, and less than half (42.3%) were predominantly breastfed in 2017 [3]. Complying with the World Health Organization’s breastfeeding recommendations can help countries meet the United Nation’s sustainable development goals with respect to good health and well-being, economic growth, and reduced inequalities [4].

Even though most mothers recognize the benefits of breastfeeding, they also face a complex web of factors that make breastfeeding difficult, including formal labor commitments without sufficient parental leave or breastfeeding support, and television ads promoting infant formula [5,6]. Over the last 30 years, the Ministry of Health in Lao PDR has attempted to increase breastfeeding rates through standard public health behavior change campaigns, including a safe motherhood program and a large United Nations children’s fund supported exclusive breastfeeding promotion campaign; however, these programs were largely unsuccessful [5].

One probable explanation for these unsuccessful interventions is the economic and financial constraints faced by mothers. A potential solution for alleviating these pressures is providing social transfers to support breastfeeding; defined as a cash or in-kind transfer. Evidence suggests that social transfers have helped increase breastfeeding rates in other settings. In a previous experiment, Puerto Rican mothers were provided US $25 per month after successful observation of breastfeeding (ie, conditional cash transfer), which resulted in significantly higher breastfeeding rates at 6 months postpartum compared with mothers who did not receive the cash transfer [7]. A similar model providing a social transfer with a letter encouraging mothers to spend time with their infant (ie, labeled social transfer) in a US neonatal intensive care unit increased the daily provision of breastmilk [8]. However, all evidence on social transfer programs for breastfeeding comes from high-income countries. In fact, no study has implemented a social transfer program for breastfeeding promotion in low- or middle-income countries despite a large number of successful social transfer programs targeting other health behaviors in these settings [9].

Capitalizing on key strategies used in previous social transfer programs, we will assess the effectiveness of social transfer programs for increasing breastfeeding rates in Vientiane, Lao PDR. A previous qualitative, community-engaged project in Vientiane focused on identifying breastfeeding norms and social transfers that would support breastfeeding mothers (unpublished data). Using the results from this study, we designed a culturally grounded social transfer program that is responsive to the identified needs of new mothers.

The overall objectives of this project are to assess (1) the effect of social transfers on exclusive and complementary breastfeeding duration and (2) the long-term impacts of breastfeeding on child development.

## Methods

### Overview

The Social Transfers for Exclusive Breastfeeding (STEB) is a prospective, parallel cluster-randomized trial in the Vientiane Capital, of Lao PDR. Mother-infant dyads will be enrolled at approximately 1 month post partum and followed for 3 years; with the ambition to extend the follow-up period. The study period runs from August 2022 to December 2026.

### Participants and Recruitment

Our study is nested within an ongoing birth cohort (the Vientiane Multigenerational Birth Cohort [VITERBI]). Women were recruited for VITERBI if they (1) lived in one of the following districts of Vientiane: Chanthabuly, Sikhottabong, Sangthong, or Mayparkngum; (2) had an expected due date/or gave birth between July 1, 2022, and June 30, 2023; (3) did not plan to permanently move outside the study area; (4) did not have a medical, intellectual, or psychological disability; and (5) agreed to participate and sign an informed consent; if younger than 18 years, a legal representative had to agree to sign the informed consent.

The target sample size for the STEB randomized control trial was 300 mothers and their children. All pregnant women enrolled in VITERBI were eligible to participate in our study if they gave birth within the last 4 weeks, were exclusively breastfeeding at the time of recruitment, had no illnesses that contraindicate breastfeeding, and had a healthy singleton infant of 37 weeks or more gestation with a birth weight of at least 2500 g.

A local study team comprised of 2 nurses and 2 laboratory technicians was responsible for enrollment and follow-up of participants. Using pregnancy records from VITERBI, a list of potential participants was created. In order to identify women who recently gave birth (≤1 month), the following data were used: (1) approximate due date or (2) weeks gestation when an approximate due date was not provided. When both variables were missing, the average week of gestation was set at 40 weeks and added to the interview date to provide the latest possible birth date. All potential participants were screened on a telephone call to ensure they met the eligibility criteria. If the participant met the inclusion criteria and agreed to participate in the study, a baseline visit was scheduled.

### Sample Size Calculation and Power

Enrolling 100 participants for each intervention arm provided us with 80% power to detect an increase in exclusive breastfeeding from the anticipated 21% in the control arm to 40% in either of the 2 intervention arms.

### Study Logistics

**Figure 1:**
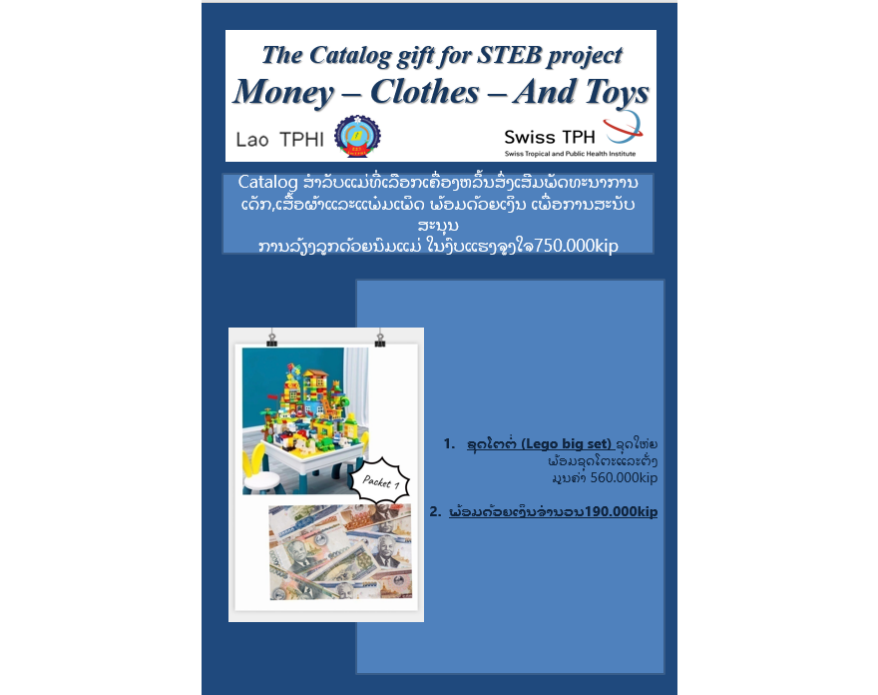
Screenshot from shopping catalog for Social Transfers for Exclusive Breastfeeding (STEB) participants.

During the baseline visit, if informed consent was given, a random number was generated within the Open Data Kit (ODK) platform that assigned the mother to a control or intervention group. If they were randomly assigned to an intervention group, mothers were allowed to choose from the following social transfers: (1) money (US $75); (2) developmental toys, clothes, and diapers for the child; or (3), a combination of money and developmental toys, clothes, and diapers for the child (Figure 1). We created a shopping catalog with different toys and clothes for them to choose from. At the end of the visit, the study team encouraged intervention group 1 to meet their breastfeeding goals and told them that the social transfer would be provided at 6 months post partum to show our support for their breastfeeding efforts. Mothers randomly selected for the third intervention arm were told that they would only receive their social transfer if they exclusively breastfed and nurses could observe breastmilk expression at the 6-month visit. All intervention and control groups received educational material about the benefits and recommendations of exclusive breastfeeding.

During a follow-up visit when the child is 6 months of age, nurses will collect an end-line survey from mothers, collect infant fecal samples, and observe breastmilk expression.

### Data Collection Instruments

#### Overview

STEB has a total of five questionnaires: (1) a screening questionnaire to assess eligibility; (2) baseline enrollment conducted at approximately 1 month post partum; (3) an endline questionnaire at 6 months post partum; (4) a follow-up visit at 1 year post partum; and (5) a follow-up visit at 2 years post partum. All source documents were created in English and translated into Lao. We briefly describe each questionnaire below.

#### Screening Questionnaire

A screening questionnaire was conducted on the telephone in order to assess the inclusion and exclusion criteria. A maximum of 10 questions were asked. If an answer to one of the questions rendered them ineligible for the study, no further questions were asked. Questions included date of birth and expected birth date in order to determine prematurity of the infant, birth parity, birthweight, current breastfeeding status, breastfeeding exclusivity, long-term medical problems of the infant, and if the mother experiences galactosemia, human immunodeficiency virus, human T-cell lymphotropic virus type 1 or 2, illicit drug use (ie, cocaine), Ebola, artificial breast implants, or infectious tuberculosis.

#### Baseline—1 Month Postpartum

The baseline questionnaire is conducted at approximately 1 month post partum and includes employment questions, the short Grit scale [10], family and gender roles, child health and vitamin, mineral or other medicine intakes, breastfeeding and infant feeding, Breastfeeding Self-Efficacy Scale–short form [11], and maternal diet. The Demographic and Health Surveys household characteristics questionnaire. Hemoglobin levels of the mother and infant were taken using the HemoCue Hb 301. Biospecimen samples included infant feces, human milk, and a saliva sample from the mother and infant. Anthropometric measurements of the child included skinfold thickness of the triceps, subscapular, quadriceps, and flank, weight, length, mid-upper arm circumference (MUAC), and head circumference. Anthropometric measurements of the mother included skinfold thickness of the triceps, subscapular, quadriceps, and flank, blood pressure, height, weight, and heart rate.

#### Endline—6 Months Postpartum

The endline questionnaire is conducted at approximately 6 months post partum and includes employment questions; child health; vitamin, mineral, or other medicine intake; breastfeeding and infant feeding; maternal diet; participation in cultural postpartum activities (eg, hotbeds and mother roasting); perceived stress scale [12]; Postpartum Specific Anxiety Scale [13]; and caregiver-reported early development instruments [14]. A variety of additional health measurements were assessed. Hemoglobin levels of the mother and infant were taken using the HemoCue Hb 301. Biospecimen samples include infant feces, human milk, and a saliva sample from the mother and infant. Anthropometric measurements of the child included skinfold thickness of the triceps, subscapular, quadriceps, and flank, weight, length, MUAC, and head circumference. Anthropometric measurements of the mother included skinfold thickness of the triceps, subscapular, quadriceps, and flank, blood pressure, height, weight, and heart rate.

#### 1- and 2-Year Follow-Up

Visits will occur at 1, 2, and 3 years post partum (SD 1 month). The questionnaire includes child health and vitamin, mineral or other medicine intake; breastfeeding and infant feeding; maternal diet; alcohol consumption; Postpartum Specific Anxiety Scale [13]; Edinburgh Postnatal Depression Scale [15]; and the brief infant sleep questionnaire–short form [16]. At 1 year post partum, child development will be assessed using caregiver-reported early development instruments [14]. At 2 and 3 years post partum, child development will be assessed using the Global Scale for Early Development [17]. Hemoglobin levels of the mother and infant will be taken using the HemoCue Hb 301. Biospecimen samples include infant feces and human milk. Anthropometric measurements of the child included foot length, skinfold thickness of the triceps, subscapular, quadriceps, and flank, weight, length, MUAC, and head circumference. Anthropometric measurements of the mother included skinfold thickness of the triceps, subscapular, quadriceps, and flank, blood pressure, height, weight, and heart rate.

### Retention of Participants

In order to reduce participant dropout, we collect a variety of contact information. Participants provide their first and family name, address, phone number, email address, village leader name, temple or religious organization, and the contact information of a family member or close friend. In addition, we provide a small incentive. Immediately after the physical examination, all participants receive the test results for both the mother and child and can track their progress over time at each visit. Test results include blood pressure, hemoglobin levels, and all anthropometric values. Results are explained in person and written on an information pamphlet. All participants who complete the baseline questionnaire receive a small gift that equates to approximately US $1.

### Outcomes

The primary end point will be exclusive breastfeeding at 6 months postpartum. Secondary end points will include exclusive and complementary breastfeeding duration, childhood wasting and stunting, child growth (height and weight), maternal and infant stress, predictors of early breastfeeding cessation, intestinal inflammation, anemia, maternal weight loss, maternal blood pressure, maternal anxiety, and GRIT personality score.

### Data Management

The ODK is used for all questionnaires and data management. All study staff have access to an Android tablet with the ODK Collect app. Data collection is completed offline on password-protected devices. Data connectivity is only used to send the final questionnaire data.

Project data will only be accessible to authorized personnel who require the data to fulfill their duties within the scope of the research project. On the case report forms and other project-specific documents, participants are only identified by a unique participant number. The unique participant number will be taken directly from VITERBI, and is a combination of the household number, district, village, and individual within the household. Health-related data will be stored for 15 years, following institutional storage and safety policies. Only samples will be kept for an unidentified amount of time if the separate consent for reuse of health data has been signed. Data storage is on a locally secured server using the ODK Aggregate server and secured via a secure sockets layer.

Biological material in this project is not identified by participant name but by a unique participant number. Biological material is appropriately stored in a restricted area only accessible to authorized personnel. The biological specimens will be stored with their code at Lao TPHI and Swiss TPH.

### Ethical Considerations

Ethical approval for the study was obtained from the Ethics Commission of Northwestern and Central Switzerland (EKNZ, 2020-00037) and from the National Ethic Committee for Health Research (044/NECHR; June 30, 2021). This research project will be conducted in accordance with the protocol, the Declaration of Helsinki [3], the Swiss Human Research Act, and the Swiss Human Research Ordinance [1], as well as other locally relevant regulations. Patients or the public were not involved in the design, conduct, reporting, or dissemination plans of our research. Eligible participants will be visited initially by study staff. The purpose of the study and its procedures will be explained to participants and written informed consent will be obtained. Pregnant women will be giving consent for themselves and their newborns. For minors (aged <18 years), both the pregnant woman and her parents will provide consent. For participants who are illiterate, there will be an additional witness who is not part of the study team. Participation is voluntary and patients have the right to withdraw from the study at any given point in time with no further obligations. If the participant withdraws at any time, all data will be deleted and biospecimen samples destroyed. Confidentiality of information will be assured to the participants. All participants younger than 18 years are considered vulnerable populations according to the Lao PDR legislation. If the respondent is underage and agrees to participate, he or she needs the authorization of a parent or legal guardian, who cosigns the study informed consent.

## Results

As of November 2023, the study has enrolled 300 participants. Study participation is ongoing until December 2027 at minimum. Over the study lifetime, 93% have completed all visits. Primary results from the 6-month visit are expected in early 2024.

## Discussion

Conditional cash transfer programs have been used in a wide range of settings to reduce poverty, with proven benefits for short- and long-term health [18]. STEB is to our knowledge the first longitudinal and randomized social transfer program evaluation for breastfeeding in a low- or middle-income country. We will follow participants for a minimum of 3 years post partum—providing an excellent platform to investigate the short- and long-term benefits of a breastfeeding social transfer program.

During the first year of the program, we encountered a variety of barriers. To start, enrolling new mothers at approximately 1 month post partum was difficult due to the high rates of infant formula use. We strategically enrolled at 1 month post partum to reduce coercion among mothers who did not want to breastfeed. However, a large majority of mothers provided infant formula before 1 month post partum. Enrolling mothers earlier in the postpartum period who want to exclusively breastfeed may reduce the early introduction of infant formula. Second, distances between the 4 study districts in Vientiane, Lao PDR are large. Transportation of staff to the rural districts is a major challenge because of the time spent traveling and the high burden of transportation costs. For example, during the rainy season, many streets become impassable—reducing the capabilities of the study staff to reach participants. Collection of infant fecal samples at 1-month was also difficult because of the irregularity of bowel movements at this age.

Despite the substantial size of the social transfers made, we believe that interventions that include financial support for breastfeeding can be highly cost-effective in this setting. In 7 Southeast Asian countries alone, the short- and long-term benefits of breastfeeding are estimated at US $1.9 billion a year [1]. We believe that social transfer programs focused on breastfeeding have the potential to be more effective in low- or middle-income countries, where monetary compensation may reduce additional barriers not present in high-income countries.

STEB has some limitations worth noting. The small sample size will prohibit more nuanced data analysis. Enrollment does not occur immediately after birth; instead, all participants are enrolled at 1-month post partum. Many mothers face significant barriers to exclusive breastfeeding during the first month postpartum; therefore, the study population of STEB may be slightly different. Recall of the exact timing of complementary foods provided is likely biased; yet, previous research has shown that recall bias for breastfeeding is minimal.

STEB was designed to provide a unique platform to investigate the short- and long-term impacts of exclusive and complementary breastfeeding on child health and development. Further, the longitudinal sampling of human milk and infant feces provides a unique opportunity to study human milk as a biological system and how this impacts the gut microbiome. If proven effective, we see potential for a long-term program that may be implemented in other low- or lower-middle-income countries with only minor modifications.

## Appendix

Multimedia Appendix 1 Peer-review report by the Swiss National Science Foundation.

## Abbreviations

Lao PDR: Lao People’s Democratic Republic
MUAC: mid-upper arm circumference
ODK: Open Data Kit
RCT: randomized controlled trial
STEB: Social Transfers for Exclusive Breastfeeding
VITERBI: Vientiane Multigenerational Birth Cohort

## Data Availability

Data sharing is not applicable to this article as no data sets were generated or analyzed during this study.
