# Supplementary material for: Social Transfers for Exclusive Breastfeeding (STEB) Intervention in Lao People’s Democratic Republic: Protocol for a Randomized Controlled Trial
Source: JMIR Res Protoc. 2024 May 3;13:e54768. doi: 10.2196/54768 (PMC11102031; doi:10.2196/54768)
Supplement: Multimedia Appendix 1 [file resprot_v13i1e54768_app1.pdf]

Review: 1

Application data

Applicant(s)
Wallenborn, Jordyn

Exclusive breastfeeding: global rates, optimal duration, and a multi-country intervention
Ambizione

Detailed evaluation

Scientific relevance, originality and topicality

The scientific relevance & topicality of the proposal are high. The influence of early life nutrition especially breastfeeding on long-term health and development is a public health priority. A revised recommendation for exclusive breastfeeding (bf) would be an important contribution to global health.

The investigator aims to test three hypotheses:

The marginal benefits of exclusive breastfeeding(bf) decline after 3-4months but remain positive even after 6 months;

Rates of exclusive bf are over-estimated globally;

Social transfers(cash in kind) to bf mothers can increase rates of exclusive bf.

Approach and methodology

Three corresponding work packages (WP) are planned.

The WP1 will be the thorough collection of evidence for optimal duration of exclusive bf.

WP2 will be global estimates of compliance with WHO recommendation (note this is different to local recommendation which in some countries including Switzerland are 4-6 months).

WP3 social transfer intervention in Brazil, based on informative qualitative research in Brazil and the pilot trial in Laos. The investigator plans to enrol 300 women to three groups which will give an 80% power to detect an increase from 21 to 40% in either of the two intervention arms of exclusive bf at 6 months.

Question- the sample size is relatively small and the hypothesised effect of doubling the rate of exclusive bf large - has the investigator considered enrolling to only two groups to increase the power of the study?

It is a worthy aim to review evidence for the WHO current recommendation of exclusive bf for 6m. As hypothesised, compliance is likely to be low and the poor evidence-base for the recommendations contributes to this. The plan for an intervention to optimise exclusive bf at 6 months may be premature until one knows the optimal duration to target (WP1). Interventions to promote bf and continued bf are important. The focus of this proposal is exclusive bf for 6m. The optimal duration of exclusive bf be different in low v high income populations. No discussion is given to any potential harm of exclusive breastfeeding such as in high income countries, possible increased rates of allergies or choosing formula.

Although the endpoint is exclusive bf to 6 months, a two year assessment of child development and growth is planned for children in the Brazil trial (WP3). Sample size is not calculated with development and growth as end points. The data can be added to that of other studies for metanalysis but is not directly relevant to the aim of determining whether social transfer intervention will increase exclusive bf at 6 m.

The investigator is already conducting a pilot trial of social transfer to improve exclusive bf at 6 months in Laos. The pilot trial is in Laos and is separately funded (76,875CHF). However, in the proposed Ambizione project, funding is requested for the assessments at 1, 2, and 3 year of age of the children of mothers recruited in the social transfer trial in Laos.

This is opportunistic and the investigator states that pooling the data from the trial in Vietnam and the one in Brazil into a multinational-pooled longitudinal database will create opportunities for future secondary research. However, assessing child development at 2-3 years of age in Vietnam and Brazil in a relatively small sample (even before loss-to-follow-up considered) will be difficult and not specifically needed to meet the aims of the proposed Ambizione project. Statistical analysis plans for follow-up data are not discussed.

Feasibility

The completion of WP 1 and 2 is highly feasible.

WP3 is challenging. The logistics of follow-up and developmental assessments of 2-3 year-olds in Vietnam and Laos will be challenging.

The investigator is well supported by her collaborators: Prof Fink and Dr Kwiatkowski from Swiss TPH, Prof Brentani from the University of Sao Paulo and Prof Kounnauong from Lao TPHI. The proposed project should strengthen links between these institutions.

Research output (publications, monographs, etc.)

The investigator is an early-career researcher who was a awarded PhD in 2017. Research output is good with 31 publications and 16 oral presentation. The investigator is a maternal and child health epidemiologist with a special interest in human milk and lactation. She has experience working in several countries. Her expertise is biostatistics. Her focus is the social factors that influence breastfeeding. She clearly wants to do more that analyse existing data. she has some experience in evaluating interventions. Her desire is lead a research group from Switzerland with a global network aimed at improving maternal and child health. She has made a good start to achieve this goal.

Specific abilities for the proposed project

The investigator states that her main contribution to publications has been data analysis and statistics. This is ideal for WP1 and WP2. Experience as a clinical trialist is more limited with the pilot study in Laos commencing January 2022. The logistics of follow-up and developmental assessments of 2-3 year-olds in Vietnam and Laos will be challenging.

The investigator is well supported by her collaborators: Prof Fink and Dr Kwiatkowski from Swiss TPH, Prof Brentani from the University of Sao Paulo and Prof Kounnauong from Lao TPHI. The proposed project should strengthen links between these institutions.

Comment

Revising and updating the evidence for WHO recommendation to exclusively breastfeed for 6 months will very likely have an impact on international guidelines. Emphasising the importance of measuring compliance with such guidelines is also essential. So WP1 and WP2 will likely have a real and significant impact.
WP3 is more innovative and more challenging. WP3 will develop the skills, expertise and networks of the chief investigator and likely establish a database for future research questions.

Note on the evaluation procedure

The evaluation bodies of the SNSF strive to reach a balanced overall assessment of each proposal. External reviews play an important role in this. Reviewers generally review only one proposal. The evaluation bodies of the SNSF, however, must compare and rate the quality of all proposals submitted by a given deadline. The opinions expressed in external reviews are generally positive, or they may occasionally include critical remarks that are largely irrelevant to the assessment conducted by the evaluation body. Therefore, the final decision taken by the SNSF evaluation bodies need not necessarily reflect the content of external reviews.

Review: 2

Application data

Applicant(s)
Wallenborn, Jordyn

Exclusive breastfeeding: global rates, optimal duration, and a multi-country intervention
Ambizione

Detailed evaluation

Scientific relevance, originality and topicality

This proposal seeks to identify the optimal duration of exclusive breastfeeding, estimate rates of exclusive breastfeeding at country, regional and global levels, and develop and test an intervention to optimise exclusive breastfeeding (social transfers). All existing social transfer programs focused on breastfeeding are currently in high-income countries. No evidence exists for low- or middle-income countries, where monetary compensation may reduce additional barriers not present in high-income countries. Low-income families are less likely to breastfeed. This project therefore provides a unique opportunity to reduce inequalities through investment in mothers and children.

The proposal is ambitious in its aims. The scientific aims of the proposal are robust, and the approaches to tackling them are generally well thought through.

The anticipated outcomes are clear. The project team covers a range of expertise needed to undertake this project, although an information scientist would add the necessary expertise to contribute specifically to work package one.

Approach and methodology

The proposal is ambitious in its aims. The scientific aims of the proposal are robust, and the approaches to tackling them are generally well thought through.

Each work package has been carefully thought through and planned. The approaches and methodology for each work package are well considered and appropriate.

Feasibility

The project team covers a range of expertise needed to undertake this project, although an information scientist would add the necessary expertise to contribute specifically to work package one.

Research output (publications, monographs, etc.)

Applicant appears to have relevant subject expertise, methodolgical expertse for the proposed project, limited previous grant application success but a significant number of outputs with impact in the research field.

Specific abilities for the proposed project

Applicant appears to have relevant subject expertise, methodolgical expertse for the proposed project, and a significant number of outputs with impact in the research field. My only concern is the applicant's limited track record of obtaining and managing grants.

Comment

this is an interesting and ambitious proposal. My only concern is that the PI has limited experience in successfully obtaining and managing grants from the information provided.

Note on the evaluation procedure

The evaluation bodies of the SNSF strive to reach a balanced overall assessment of each proposal. External reviews play an important role in this. Reviewers generally review only one proposal. The evaluation bodies of the SNSF, however, must compare and rate the quality of all proposals submitted by a given deadline. The opinions expressed in external reviews are generally positive, or they may occasionally include critical remarks that are largely irrelevant to the assessment conducted by the evaluation body. Therefore, the final decision taken by the SNSF evaluation bodies need not necessarily reflect the content of external reviews.

Review: 3

Application data

Applicant(s)
Wallenborn, Jordyn

Exclusive breastfeeding: global rates, optimal duration, and a multi-country intervention
Ambizione

Detailed evaluation

Scientific relevance, originality and topicality

This is an important, timely topic, and the candidate has laid out a robust and original approach to pursuing new information about a more accurate assessment of the prevalence of exclusive breastfeeding (EBF), as well as for testing impacts of an intervention to increase EBF.

Approach and methodology

Jordyn has laid out a thoughtful and rigorous approach to answering each of her three research questions.

Feasibility

The combination of Jordyn's expertise, the existing datasets, and the collaborations to assess the impact of an intervention in 2 very different settings bodes well for a highly feasible project.

Research output (publications, monographs, etc.)

(FYI: The following responses were originally much longer, but the website ate my first set of answers.)

Jordyn has a publication/presentation record, that is very impressive for her career stage, both in terms

of quality and quantity. It looks like the number of peer reviewed publications are rapidly increasing across time; I am excited to see what comes next.

Specific abilities for the proposed project

Jordyn's training in human biology and robust quantitative epidemiological techniques poises her to achieve all that she has set out in this application. She also has ample exposure to public health more broadly considered. The results about the role of EBF in human health and the utility of the interventions for prolonging EBF will be of broad interest in public health and policy domains.

The evolution of Jordyn’s career suggests that she continues to expand her skillset, independently of any one single advisor or department. The project she lays out here indicates to me that she is a creative thinker who can significantly advance the field of infant feeding.

Comment

Her proposal is exceedingly well put together, of both scientific and policy interest, she is well-trained to achieve this research and will be supported by a strong international team of high-caliber researchers.

Note on the evaluation procedure

The evaluation bodies of the SNSF strive to reach a balanced overall assessment of each proposal. External reviews play an important role in this. Reviewers generally review only one proposal. The evaluation bodies of the SNSF, however, must compare and rate the quality of all proposals submitted by a given deadline. The opinions expressed in external reviews are generally positive, or they may occasionally include critical remarks that are largely irrelevant to the assessment conducted by the evaluation body. Therefore, the final decision taken by the SNSF evaluation bodies need not necessarily reflect the content of external reviews.

Review: 4

Application data

Applicant(s)
Wallenborn, Jordyn

Exclusive breastfeeding: global rates, optimal duration, and a multi-country intervention
Ambizione

Detailed evaluation

Scientific relevance, originality and topicality

This project on global rates and optimal duration of exclusive breastfeeding is well-prepared and has public health and clinical relevance. It is proposed to a) investigate the relationship between exclusive breastfeeding duration and child health and development, b) to create comparable updated estimates for compliance with recommendations at local and international levels, and c) to implement and test an intervention to optimize exclusive breastfeeding in two LMIC with low exclusive breastfeeding rates in Southeast Asia (Lao PDR) and South America (Brazil).
A study will be carried out to build a large, multi-country database pooling international birth cohort studies to identify the optimal duration of exclusive breastfeeding for child health and development. In complement to that, an experimental study will test the efficacy of a social transfer intervention for increasing exclusive breastfeeding rates in both LMIC mentioned above. The results generated by this project will provide a) evidence on the optimal duration of exclusive breastfeeding and b) a global assessment of where countries are relative to this optimal duration; c) the creation of a new, multinational-pooled longitudinal database that will advance our understanding of main barriers for breastfeeding in LMIC.
The strengths of the project are represented by the profile of the applicant (independent young scientist focused and experienced in the specific research field and providing academic mobility), and the high level of collaboration among different specialties and (above all) with the institutions on both sites abroad.
However, while the creation of a multi-country database on breastfeeding (WP1) is original and needed, the other research parts (WP2, WP3) lack of the same level of originality and the intervention study does not provide a close link to the other project parts (e.g. does not test the evidence originating from WP1 and WP2).

Approach and methodology

WP1: Based on the fact that human milk feeding is increasingly recognized as critical to the development of the immune system and other health and neurodevelopmental outcomes in offspring, the applicant aims to provide new, state-of-the-art evidence on optimal duration of exclusive breastfeeding. The approach that pools datasets from international birth cohort studies and intervention studies, and uses Bayesian statistics is well described and original. However, the targeted outcomes and the precise meaning of a related ‘optimal’ (occasionally ‘ideal’) duration of breastfeeding are not described in detail. Furthermore, given the ethical constraints that limit (for obvious reasons) any random assignment of a newborn to the exclusive own mother milk feeding, the methods section would have benefited from a brief but clear statement on the limits of observation studies (and related meta-

analyzes) reporting cause-and-effect relationships between human milk and offspring outcomes (e.g. potential / unmeasured confounding psycho-social factors).
WP2: Based on large and representative (international) databases, the applicant aims also to create national, regional, and global estimates of compliance with the current WHO exclusive breastfeeding recommendation (6 months). In this WP, the approach is similar as that described in WP1, where international datasets on characteristics of breastfeeding are assembled and classical epidemiology and Bayesian statistics is used. The approach is clearly described and the method is valid.
WP3: Based on an already planned intervention study in an LMIC (STEB in Lao PDR), the applicant will initiate a new, culturally adapted intervention study in Brazil, in which mothers will be randomly assigned to 1) a control group without intervention, 2) a first intervention arm with receipt of a social transfer at the baseline and 3) a second intervention arm with reception of the social transfer at the baseline plus a second social transfer conditional on exclusive breastfeeding at the 6-month visit. The final aim of the applicant is to validate and implement a multi-country intervention to increase exclusive breastfeeding. The description of the qualitative (informative) and organization (logistic) aspects of the study is sound. The intervention (social transfer) is well-described but some peculiar characteristics of an intervention study are missing (conduction). Additionally, while the primary outcome of the intervention is the increase (occasionally ‘optimize’) of exclusive breastfeeding, it is not clear how any observed relationships between the intervention (e.g. culturally grounded social transfer for participating mothers in form of gift vouchers, food or child books) and offspring outcomes will be interpreted.

Feasibility

The project is ambitious but based on the applicant's qualification, her experience and commitment in the field, and the collaborative network is feasible and promising (data acquisition and analysis):
- The applicant provides the necessary skills to independently initiate and conduct the proposed study project and to successfully achieve the defined objectives.
- The candidate provides excellent experience in the area of breastfeeding research (relevant output profile and overall record in the implementation of breastfeeding program and its impacts on maternal and infant health).
- The applicant provides very good experience in biostatistics and epidemiology methods.
- The host institution (TPH) and the collaborative sites in Lao PDR and Brazil have already made available important resources for the success of the applicant’s project.

Research output (publications, monographs, etc.)

Scientific quality of the research output
Over the past 10 years, the applicant has been very productive and has provided her research output in peer-reviewed journals (24 articles to date: 21 as first and 4 as last author, total h-index 11 and i10-index 14)with constantly increasing quality. The applicant has been also an active presenter (poster and oral) at important international conferences (e.g. Swiss Public Health Conference, World Congress on Public Health, American Public Health Association) and has been awarded with several competitive junior investigator awards/grants at every stage of applicant's career.
Research output at the various career stages
- Doctoral period and prior: very high rate of peer-reviewed publications as first author in international Journals with focus on lactation and maternal health.
- Post-doctoral period: same high level of scientific productivity with increasing number of papers as last author and differentiation/expansion of the target Journals (public health, pediatrics).
Productivity (that is, qualitative output in relation to net academic age)
In relation to her net academic age, the applicant provided scientific output of increasingly high quality (in terms of methodology and public health relevance).

Scientific independence
Based on the number of publications as last author and the outreach activities, the scientific independence of the applicant has significantly increased over the past 5 years.
Impact in the research field
The research work of the applicant and the its impact in the specific research field of breastfeeding and maternal health has been significant (e.g. evaluation/implementation of breastfeeding promotion programs).

Specific abilities for the proposed project

The applicant provides the needed competences to start and to lead this project, based on the following considerations:
- The applicant provides excellent education / training in maternal and child health epidemiology, gained over the past 12 years in the United States (North Dakota State University, Virginia Commonwealth University, University of California Berkeley) and in Switzerland (and University of Basel).
- The academic mobility of the candidate is excellent, starting with her first training activity and continuing throughout her doctoral and postdoctoral careers.
- The level of expertise in the specific field of the proposed project is excellent with a clear focus of research on breastfeeding and related maternal and offspring outcomes.
- The previous research work and commitment to promoting maternal and child health in LMICs (where a relevant part of the project is foreseen) and the level of independence as scientist reached in the last 5 years reinforce the candidate's profile for the success of this project.

Comment

This is a very good research project of clinical and health relevance, submitted by a young applicant with an excellent scientific profile. Building on the accurate extraction of data from aggregated datasets from international birth cohorts and trials and supported by a robust statistical approach, the results generated by this project will provide evidence on (WP1) optimal duration of exclusive breastfeeding and (WP2) a global assessment of where countries are relative to this optimal duration. Furthermore, (WP3) a social transfer to improve the duration of exclusive breastfeeding in two LMICs will be implemented and tested through an intervention study. Although the targeted creation of a multinational database on breastfeeding and related outcomes (in mothers and their offspring) is original and necessary, the other parts of the research lack the same level of originality and the intervention study does not provide a close link with the other parts of the project. Aside from the lack of minor methodological aspects, the study approach and its statistical plan are valid. The project is supported by a consolidated international collaboration network (Switzerland-Lao PDR-Brazil) which further increases the chances of success.

Note on the evaluation procedure

The evaluation bodies of the SNSF strive to reach a balanced overall assessment of each proposal. External reviews play an important role in this. Reviewers generally review only one proposal. The evaluation bodies of the SNSF, however, must compare and rate the quality of all proposals submitted by a given deadline. The opinions expressed in external reviews are generally positive, or they may occasionally include critical remarks that are largely irrelevant to the assessment conducted by the evaluation body. Therefore, the final decision taken by the SNSF evaluation bodies need not necessarily reflect the content of external reviews.

Review: 5

Application data

Applicant(s)
Wallenborn, Jordyn

Exclusive breastfeeding: global rates, optimal duration, and a multi-country intervention
Ambizione

Detailed evaluation

Scientific relevance, originality and topicality

The project focus on a very important topic. Breastfeeding is a key strategy to maximize the health and the human capital of both mothers and children. Efforts to better understand its current status and to propose strategies to improve levels of breastfeeding in the population are very welcome.

The project is organized in three work packages. The first aims to estimate the optimal breastfeeding duration, the second will estimate country, regional and global rates of exclusive breastfeeding using DHS and MICS surveys, and the third is an intervention aiming to increase the proportion of mothers exclusively breastfeeding during the first six months of life of the baby.

The project is generally superficial, and I am not sure if it is due to a limitation in the size of the proposal. But in the three packages the definition of outcomes and procedures is insufficient for a more thorough assessment of the project.

Having said that, it is not easy to assess relevance. If all goes as planned and proposed the project will be a turning point in the breastfeeding field. All objectives are extremely relevant, but I have a few concerns.

The estimation of optimal duration is a complex task given this optimal duration will change depending on the outcome in question and on the setting.

Using surveys to estimate prevalence of exclusive breastfeeding has limitations that I could not identify in the text how they will be dealt with.

And regarding the proposed intervention that focus on "social transfers", apparently cash, the proposal seems to ignore the extensive literature of barriers to breastfeeding. That is actually surprising given the academic record of the applicant. Barriers range from commercial determinants, including the intensive marketing of baby food products, to social perceptions, work limitations, down to individual situations of the woman and the baby. At least in Brazil, the lack of economic resources does not seem to be a leading barrier to breastfeeding.

Approach and methodology

WP1 - Estimation of optimal breastfeeding duration

Adding evidence of breastfeeding benefits is always welcome. However, stating this objective in a general way may be misleading since the effects of breastfeeding may be different for different outcomes (e.g. growth or development) and change across settings. It is unlikely that this single analysis will be enough to change what WHO proposes presently.

“We will specifically focus on low- and middle-income countries due to the critical need in these settings to optimize breastfeeding practices”

This seems to imply that breastfeeding is more important in poor than rich countries, when it is important everywhere. Also, the impact of duration of breastfeeding may be different in rich and poor countries what might justify separating settings when exploring this effect.

The outcomes are loosely defined through a list of variables and it is not possible to know at what age each of the outcomes will be evaluated.

The statistical analysis proposed seems promising but running this type of analysis on pooled data is always a challenge given the different ways outcomes and confounders are measured. Also, it would be interesting to analyse longer periods of exclusive breastfeeding – is there a moment when it is not beneficial anymore?

WP2 - Estimation of compliance with WHO recommendation

There is ample literature on breastfeeding duration using DHS and MICS surveys. Other sources, including the birth cohorts mentioned in the previous, may offer better opportunities to estimate the proportion of children that are exclusively breastfed until six months (or other ages). The problem with standard survey data is that, as the aplicant mentioned in the background section, we just have information on whether the children received only breastmilk the day before the interview. If the child is not being exclusively breastfed, there is no information as to the duration of exclusive breastfeeding. It is not clear from the statistical analysis section what will be done to circumvent this limitation.

Updating the estimation of data availability, there are 120 countries and 410 surveys DHS or MICS from 1993 to 2020 where exclusive breastfeeding can be estimated.

WP3 – Multi-country intervention to increase exclusive breastfeeding based on cash transfers

The proposed intervention is interesting but I wonder what further impact this could have in Brazil where a large proportion of the poor population is already receiving monthly payments of R$ 400. It is surprising that the intervention does not include any type of information or support. Also, the proof of breastfeeding at the end of the 6-month period – milk at breast expression – seems to me degrading, to say the least. And it does not prove exclusive breastfeeding or breastfeeding at all.

- Informative research

  It is not clear what is the objective of this. The proposal states “identification of culturally appropriate social transfers for breastfeeding”, but next section presents an estimate of how much would cover the “cost” of breastfeeding. It seems to me that exploring barriers to breastfeeding would be more productive.

- Social transfers

  This section seems to ignore that employed women in Brazil are covered by a 4-month maternity leave with full pay. Also, it seems to ignore the existing cash transfer programs that will inevitably overlap with this study. The choice to be given to people sounds naïve – I have little doubt that cash will be the choice of people every time.

- Logistics, sample size and evaluation

Enrolling women who gave birth in the previous month may be too late. Many mothers will have had stopped breastfeeding or are already partially breastfeeding. How will the study minimize losses to follow-up? After six months many of the women will have moved or changed their telephone number.

Recruiting only women that are exclusively breastfeeding within the first month after birth can severely bias the sample towards mothers that are already inclined to breastfeed. In many cases, other milks are introduced in the first few days of the baby's life due to a variety of reasons.

The sample size does not allow for losses of follow-up and does not provide any specific parameters used for the calculation. It sounds terribly optimistic to me.

Adding to the optimism, nothing is said about how stools collection will be conducted. This is not a simple matter even with babies. It will entail a visit to the family, correct collection, and a return visit to collect the sample.

Feasibility

Given the experience of the applicant, I do not see any great difficulties in taking the project to a successful end. As mentioned above, some strategies to limit or allow for follow-up losses will be necessary. This is not an unsurmountable problem given the relatively small size of the intervention sample.

Work packages 1 and 2 are based on secondary data. The difficulty with WP1 will be the time needed to put together all the data. Contacting investigators and getting the data takes considerable time. Otherwise, I do not foresee any other difficulties apart form harmonizing the data.

Research output (publications, monographs, etc.)

The applicant finished her PhD less than five years ago and already amasses a considerable research output, with more than 30 papers published in peer reviewed journals. Her work has been published in high profile journals and has received a fair number of citations.

The focus of her work is very clear, with publications on child health and development, mostly related to breastfeeding. She has studied both high and middle/low income countries.

She has also worked and participated in several different institutions. She seems very promising in terms of career.

Specific abilities for the proposed project

Her previous experience in breastfeeding research is key to the success of the present proposal. She has

extensive knowledge of the issues related to breastfeeding and its advantages and benefits. She has very good previous field work experience.

Comment

The project touches very relevant aspects of breastfeeding and if successfully completed can bring important advances to the field. However I have concerns related to lack of details in key aspects of the project. Also, an intervention aimed at incentivizing breastfeeding that uses only transfers seems rather limited. Again, it seems that more details could help.

Note on the evaluation procedure

The evaluation bodies of the SNSF strive to reach a balanced overall assessment of each proposal. External reviews play an important role in this. Reviewers generally review only one proposal. The evaluation bodies of the SNSF, however, must compare and rate the quality of all proposals submitted by a given deadline. The opinions expressed in external reviews are generally positive, or they may occasionally include critical remarks that are largely irrelevant to the assessment conducted by the evaluation body. Therefore, the final decision taken by the SNSF evaluation bodies need not necessarily reflect the content of external reviews.
